# Supplementary material for: Deletion of LRP1 in Smooth Muscle Cells Induces Infrarenal Aortic Aneurysms in Mice
Source: bioRxiv. 2026 Oct 22:2026.10.16.759442. Preprint. [Version 1] doi: 10.64898/2026.10.16.759442 (PMC13738480; doi:10.64898/2026.10.16.759442)
Supplement: Supplementary file 1 [file media-2.pdf]

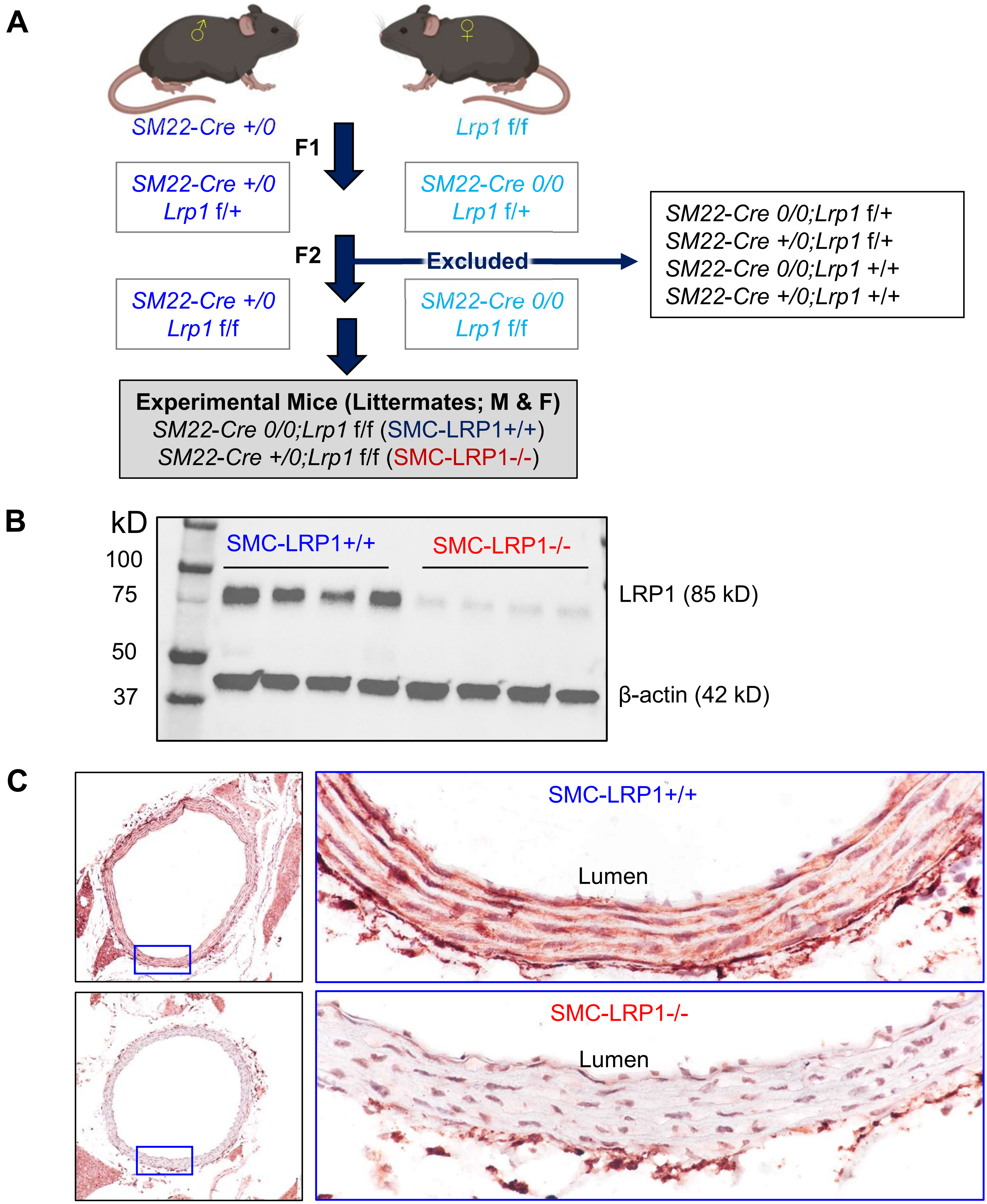


**Figure S1. Breeding strategy and confirmation of LRP1 deletion in smooth muscle cells (SMCs). (A)** Breeding strategy to develop SMC-LRP1+/+ and SMC-LRP1-/- littermates. LRP1 deletion in aortic SMCs was confirmed by Western blot **(B)** and immunostaining of aortas **(C)** in SMC-LRP1-/- mice. Graphic mouse images were created with BioRender.

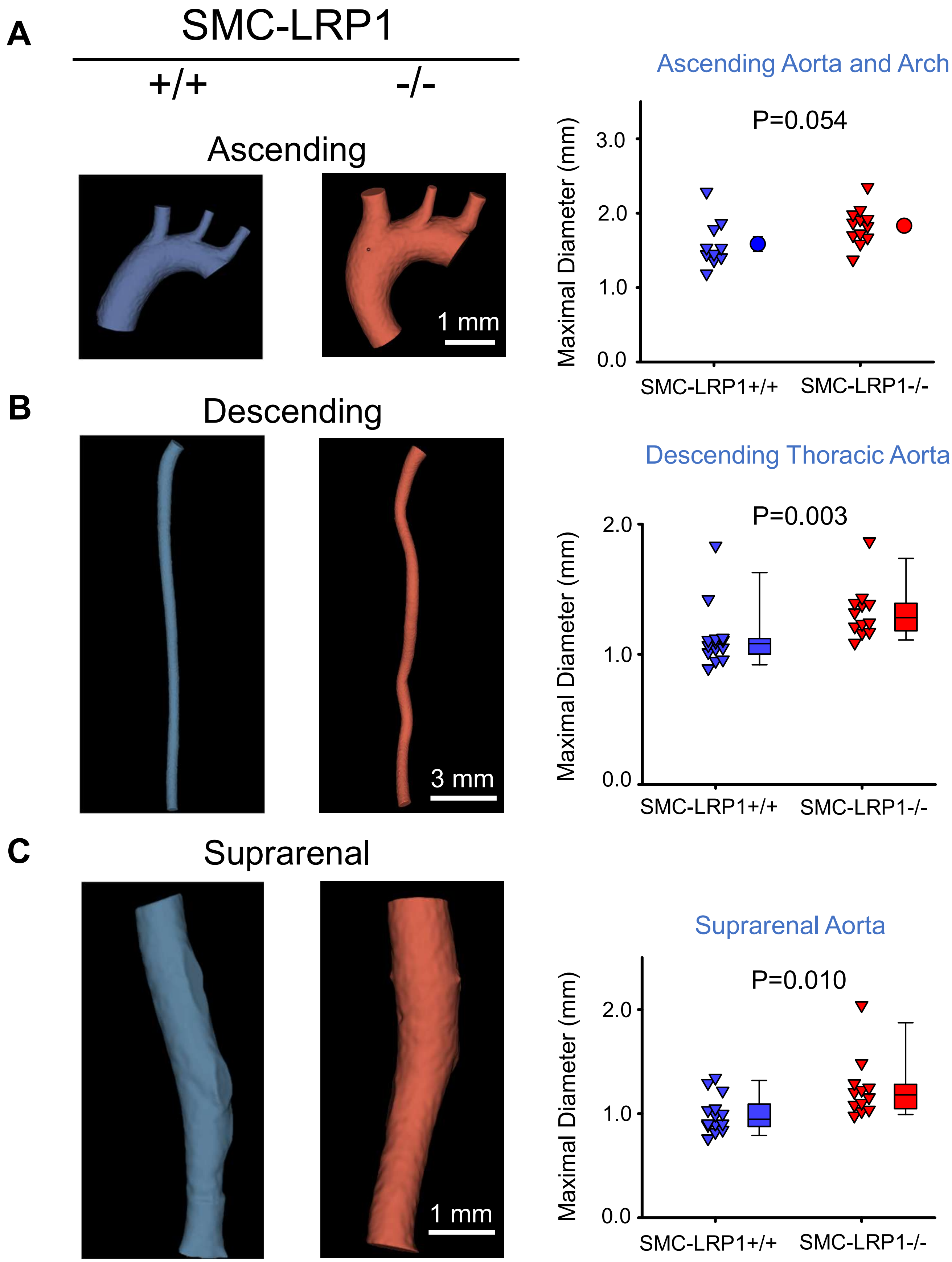


**Figure S2. LRP1 deletion in SMCs induced multiple aortic pathologies spontaneously in male mice.** 3D reconstruction of micro-CT images and diameter quantification of the ascending **(A)**, descending thoracic **(B),** and suprarenal **(C)** regions in male SMC-LRP1+/+ and SMC-LRP1-/- mice at 40 weeks of age. N =10 to 14/group. Statistical analysis: Student's t test (**A**) or Mann-Whitney rank sum test (**B-C**).

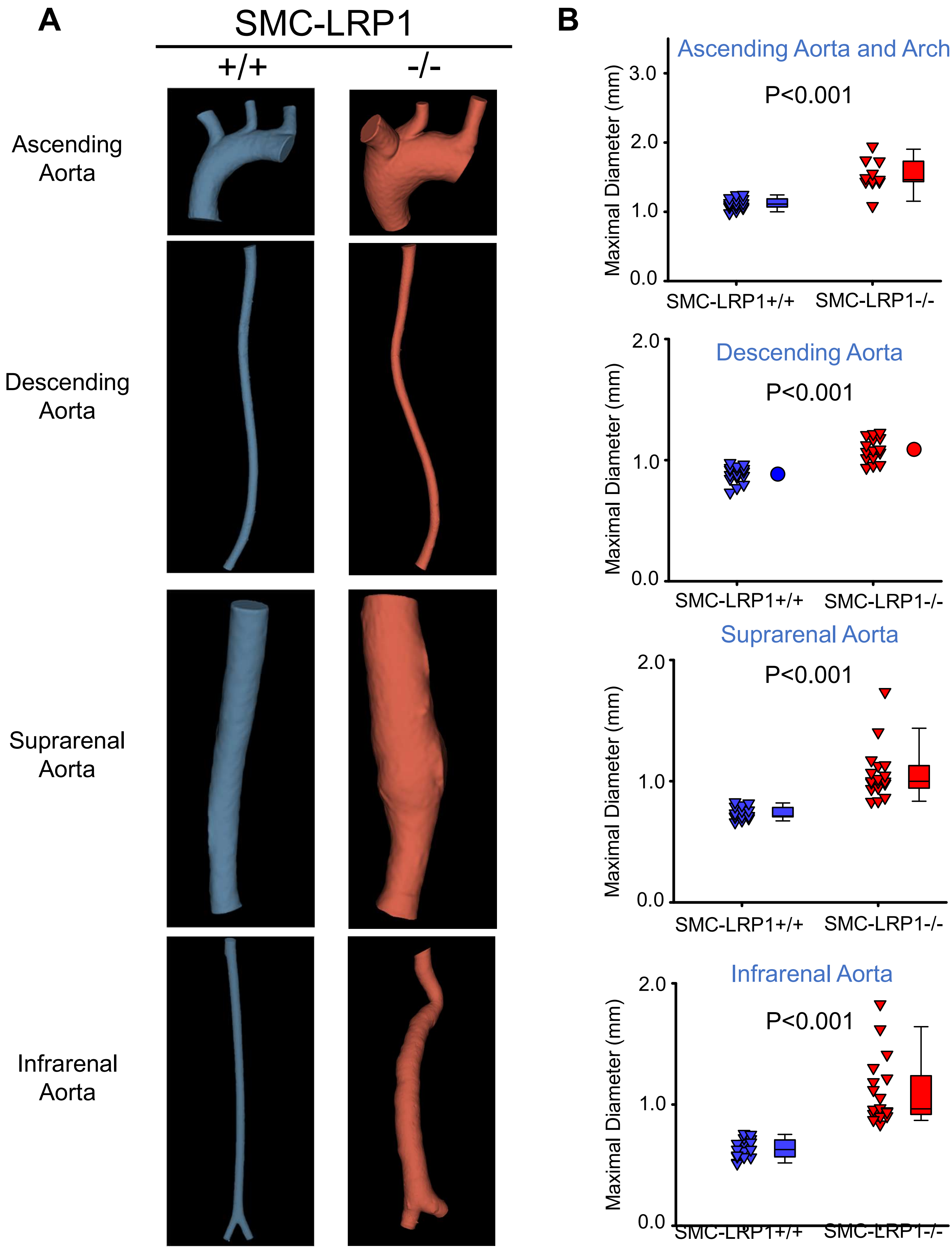


**Figure S3. LRP1 deletion in SMCs induced multiple aortic pathologies spontaneously in female mice.** 3D reconstruction of micro-CT images **(A)**, and diameter quantification **(B)** of the ascending, descending, suprarenal, and infrarenal regions in female SMC-LRP1+/+ and SMC-LRP1-/- mice at 40 weeks of age. N=14 to 16/group. Student's t test (descending thoracic aorta) or Mann-Whitney rank sum test (other aortic regions).

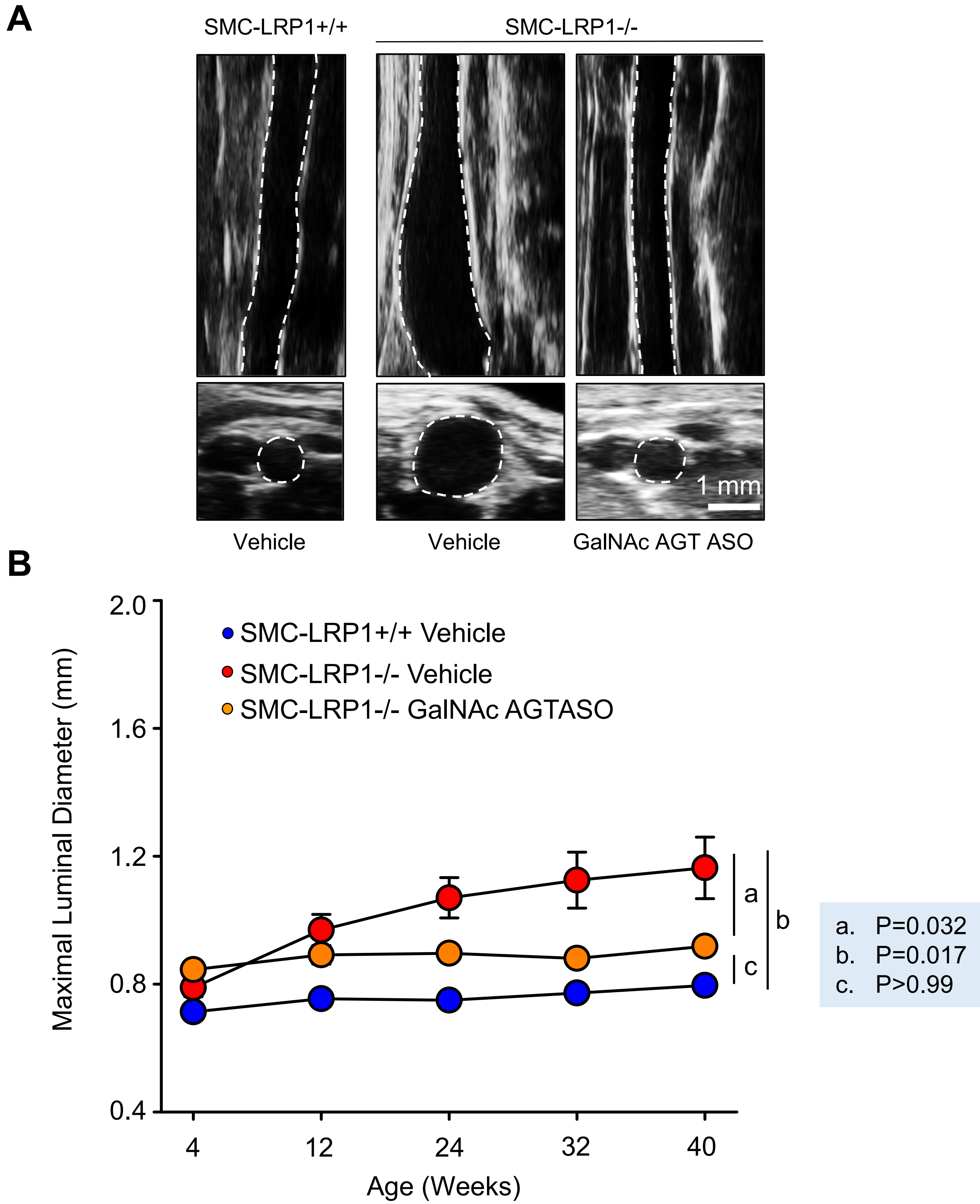


**Figure S4. Progressive infrarenal aortic luminal dilatations in female SMC-LRP1-/- mice were attenuated by GalNAc AGT ASO.** Representative ultrasound images of infrarenal aortas from 40-week-old SMC-LRP1+/+ and SMC-LRP1-/- mice **(A)**. Ultrasonographic measurements of infrarenal aortic luminal diameters from 4 to 40 weeks of age in SMC-LRP1+/+ and SMC-LRP1-/- female mice administered vehicle or GalNAc AGT ASO. N = 9-10/group **(B)**. Statistical analysis was performed using a mixed-effects model with random intercepts and random slopes for time (week).
